# Clinical and angiographic characteristics of very young patients undergoing invasive coronary angiography

**DOI:** 10.3389/fcvm.2026.1849565

**Published:** 2026-08-31

**Authors:** Xing Hu, Xuefeng Wang, Kerui Huang, Yi Zhong, Yong Luo, Ji Li, Yuanmin Hu

**Affiliations:** 1 Department of General Practice, The Affiliated Hospital, Southwest Medical University, Luzhou, Sichuan, China; 2 Department of Cardiology, The Affiliated Hospital, Southwest Medical University, Luzhou, Sichuan, China; 3 Department of Interventional Medicine, The Affiliated Hospital, Southwest Medical University, Luzhou, Sichuan, China; 4 College of Basic Sciences, Southwest Medical University, Luzhou, Sichuan, China; 5 Department of Health Management Center, The Affiliated Hospital, Southwest Medical University, Luzhou, Sichuan, China

**Keywords:** atherosclerosis, coronary angiography, coronary artery disease, risk factor, young

## Abstract

**Background:**

Atherosclerotic risk factors are increasingly prevalent among younger populations, contributing to a rise in premature coronary artery disease. This study aims to investigate the clinical and angiographic characteristics of this specific demographic.

**Methods:**

This retrospective study enrolled 215 consecutive patients aged 35 years or younger (181 men) who underwent coronary angiography (CAG) at our center. Clinical data and angiographic images were collected and analyzed. Independent predictors of ischemic heart disease (IHD) were evaluated using logistic regression analysis. Discriminative performance was assessed using receiver operating characteristic curve analysis.

**Results:**

In the selected cohort of 215 young patients, the prevalence rates of obesity/overweight, smoking, dyslipidemia, hypertension, and diabetes mellitus were 56.7%, 53.0%, 31.2%, 23.3%, and 8.4%, respectively. Within this cohort, 92 (42.8%) patients exhibited various forms of IHD, with 85.9% of cases attributed to atherosclerotic cause and 14.1% attributed to nonatherosclerotic etiologies. A total of 202 (94.0%) patients underwent CAG due to chest pain, ischemic electrocardiogram changes, and/or elevated high-sensitivity cardiac troponin T (hs-cTn T) levels. IHD prevalence was highest at 73.8% for those with all three indications, compared to 44.1% with two, and 9.2% with one or none (all *p* < 0.001). Multivariate logistic regression analysis identified diabetes mellitus (odds ratio [OR], 14.71; 95% confidence interval [CI], 1.59-135.76; *p* = 0.018), ischemic electrocardiogram changes (OR, 3.11; 95% CI, 1.09-8.91; *p* = 0.034), interventricular septum thickness (OR, 1.46; 95% CI, 1.08-1.98; *p* = 0.015), and elevated hs-cTn T levels (OR, 8.26; 95% CI, 3.17-21.51; *p* < 0.001) as independent predictors of IHD. The combined multivariable model demonstrated a robust discriminative performance (AUC, 0.85; 95% CI, 0.79-0.90; *p* < 0.001).

**Conclusions:**

The present study demonstrated a significant prevalence of modifiable atherosclerotic risk factors among very young patients undergoing CAG. Although atherosclerosis was identified as the primary cause of IHD, nonatherosclerotic etiologies also contributed substantially. The presence of two or more angiographic indications—namely, chest pain, ischemic electrocardiogram changes, and/or elevated hs-cTn T levels—was associated with an increased risk of IHD. Furthermore, diabetes mellitus, ischemic electrocardiogram changes, interventricular septum thickness, and elevated hs-cTn T levels were independent predictors of premature IHD.

## 1 Introduction

Atherosclerotic cardiovascular disease (ASCVD) is one of the leading causes of mortality globally (1). Although it primarily affects older individuals, there has been a concerning rise in cases of premature acute myocardial infarction (AMI) in recent years (2). Traditional risk factors for ASCVD, such as hypertension, diabetes, dyslipidemia, obesity, smoking, and physical inactivity, are increasingly prevalent among young adults and even children (2–4). Furthermore, emerging lifestyle factors, including high stress levels, sleep disturbances, prolonged television viewing, and poor dietary habits, have been identified as additional risk contributors (5–8).

Notably, various nonatherosclerotic coronary artery disorders, including coronary spasm, coronary thrombosis, and congenital coronary artery anomalies, tend to affect younger individuals and can lead to ischemic heart disease (IHD) and even sudden cardiac death (9–12). Additionally, conditions such as myocarditis, cardiomyopathy, psychosomatic disorders, and gastropathy can mimic IHD, presenting significant diagnostic challenges and complicating treatment strategies (13, 14).

The consequences of missed or misdiagnosed premature AMI can be significant both clinically and socially. Nonetheless, there is a paucity of data characterizing very young patients undergoing invasive coronary angiography (CAG). Therefore, this study aims to investigate the clinical and angiographic characteristics of this specific demographic.

## 2 Materials and methods

### 2.1 Study population

This study employed retrospective design. Consecutive patients aged ≤ 35 years undergoing invasive CAG at the Affiliated Hospital of Southwest Medical University between September 2018 and October 2024 were included. Exclusion criteria included suboptimal CAG images. Electronic medical records were retrospectively reviewed to analyze the clinical features of the patients. Additionally, all CAG images were extracted and independently analyzed through visual estimation by two experienced interventional cardiologists (Y.L. and Y.Z.) who were blinded to the patients' profiles; any discrepancies were resolved by a third cardiologist (X.W.).

### 2.2 Ethical approval and consent

This study was conducted according to the principles of the Declaration of Helsinki and was approved by the ethics committee of our institute, which waived the requirement for informed consent due to the retrospective nature of the research.

### 2.3 Definitions

#### 2.3.1 Ischemic electrocardiogram change

Ischemic electrocardiogram (ECG) change was diagnosed if a patient's ECG showed any of the following criteria: (1) pathological Q waves; (2) ST-segment elevation [at least two contiguous leads with ST-segment elevation ≥ 2.5 mm in men < 40 years, ≥ 2 mm in men ≥ 40 years, or ≥ 1.5 mm in women in leads V2–V3 and/or ≥ 1 mm in the other leads (in the absence of left ventricular hypertrophy or left bundle branch block)]; (3) ST-segment depression (≥ 1 mm); or (4) T-wave inversion.

#### 2.3.2 Ischemic heart disease

Ischemic heart disease (IHD) was defined as the presence of chest pain or other symptoms suggestive of ischemia, corroborated by angiographic evidence of myocardial ischemia. This condition includes atherosclerotic cause resulting in a narrowing of ≥ 50% in one or more major epicardial coronary arteries (coronary artery disease, CAD), as well as nonatherosclerotic causes such as coronary thrombosis, inflammation, or spontaneous coronary artery dissection leading to similar arterial narrowing. Additional nonatherosclerotic etiologies include slow coronary blood flow (defined as one or more major epicardial coronary arteries with a corrected thrombolysis in myocardial infarction frame >27) (15), severe coronary spasm (defined as focal or diffuse luminal narrowing of ≥70% in one or more major epicardial coronary arteries, which could be reduced to less than 50% following the intracoronary administration of nitroglycerin or sodium nitroprusside), and severe myocardial bridging (defined as luminal narrowing of ≥ 50% during systole in one or more major epicardial coronary arteries).

#### 2.3.3 Acute myocardial infarction

AMI was defined as the presence of myocardial necrosis in a clinical setting consistent with myocardial ischemia (14).

#### 2.3.4 Normal coronary artery

A normal coronary artery was defined as angiographically smooth vessels, free from plaques, stenosis, dilation, and anomalies. Furthermore, the coronary blood flow in all coronary arteries is classified as Thrombolysis in Myocardial Infarction (TIMI) grade 3.

#### 2.3.5 Valvular heart disease

Valvular heart disease was defined as moderate or severe valvular regurgitation and/or stenosis.

### 2.4 Statistical analysis

Statistical analyses were conducted using MedCalc Software, version 23.4.1 (MedCalc Software Ltd, Ostend, Belgium). Continuous variables with a normal distribution were reported as mean ± standard deviation (SD) and compared using Student's t-test. Non-normally distributed variables were expressed as median and interquartile range (IQR) and compared using the Mann–Whitney U test. Categorical variables were presented as counts and percentages, with differences between groups examined using Pearson's chi-squared test or Fisher's exact test.

Variables showing statistical significance in univariate analysis (*p* < 0.05), along with clinically relevant parameters, were included in a multivariable logistic regression model to identify independent predictors of IHD. The strength of associations was expressed as odds ratios (ORs) and corresponding 95% confidence intervals (CIs). Receiver operating characteristic (ROC) curve analysis was used to evaluate the discriminative performance of combined multivariable model. All *p-*values were two-tailed, and a *p*-value < 0.05 was considered statistically significant.

## 3 Results

### 3.1 Baseline characteristics

A total of 215 eligible patients were included in this study. Their demographic and clinical characteristics are presented in Table 1. The median age at the time of CAG was 32 years (ranging from 13 to 35 years), with 181 (84.2%) patients being male.

**Table 1:** Demographic and clinical characteristics of very young patients undergoing coronary angiography.

Characteristics	Overall (*N* = 215)
Age, y	32 (29–34)
Male, *n* (%)	181 (84.2)
BMI, Kg/m^2^	25.5 ± 4.6
Smoking, pack.year	1 (0–10)
Clinical manifestations, *n* (%)	
Chest pain	159 (74.0)
Dyspnea	116 (54.0)
Palpitation	68 (31.6)
Asymptomatic	4 (1.9)
Comorbidity, n (%)	
Hyperuricemia	92 (42.8)
Dyslipidemia	67 (31.2)
Hypertension	50 (23.3)
Diabetes mellitus	18 (8.4)
Valvular heart disease	7 (3.3)
Prior coronary artery disease	7 (3.3)
Electrocardiographic findings, *n* (%)	
ST-segment elevation	89 (41.4)
T-wave inversion	68 (31.6)
Pathological Q waves	52 (24.2)
ST-segment depression	35 (16.3)
Arrhythmia	24 (11.2)
Left ventricular hypertrophy	10 (4.7)
Echocardiographic findings	
LA dimension, mm	31 (29–35)
LV dimension, mm	48 (44–52)
RA dimension, mm	42 (40–45)
RV dimension, mm	20 (19–22)
IVS thickness, mm	10 (9–11)
LVPW thickness, mm	10 (9–10)
LVEF, %	64 (58–66)
Moderate or severe valvular regurgitation, *n* (%)	7 (3.3)
Moderate or severe valvular stenosis, *n* (%)	1 (0.5)
Laboratory tests	
Hemoglobin, g/L	151 (141–160)
LDL-C, mmol/L	2.8 (2.2–3.5)
Serum creatinine, mmol/L	70.4 (61.6–81.3)
Serum uric acid, mmol/L	409 (325–479)
Peak NT-pro BNP, pg/mL	61.5 (34.5–511)
Peak hs-cTnT, pg/mL	0.04 (0.006–1.38)

BMI, body mass index; LA, left atrium; LV, left ventricle; RA, right atrium; RV, right ventricle; IVS, interventricular septum; LVPW, left ventricular posterior wall; LVEF, left ventricular ejection fraction; LDL-C, low-density lipoprotein cholesterol; NT-pro BNP, N-terminal pro-B-type natriuretic peptide; hs-cTnT, high-sensitivity cardiac troponin T.

In the present study, the most prevalent symptoms observed among the patients were chest pain, dyspnea, and palpitations. Additionally, a significant proportion of the patients (78.1%) exhibited at least one traditional ASCVD risk factor. Among these, obesity/overweight (56.7%), smoking (53.0%), dyslipidemia (31.2%), hypertension (23.3%), and diabetes mellitus (8.4%) were prevalent. Notably, ischemic ECG change was present in 145 (67.4%) patients, and ST-segment elevation was observed in 89 (41.4%) patients, of whom 65 (30.2%) received a diagnosis of acute ST-segment elevation myocardial infarction (STEMI). Most patients exhibited preserved left ventricular ejection fraction (LVEF ≥ 50%) on echocardiography, with only 9 (4.2%) patients showing a mildly reduced LVEF (41-49%) and 11 (5.1%) patients exhibiting reduced LVEF (≤ 40%). Furthermore, high-sensitivity cardiac troponin T (hs-cTn T) levels were elevated in 117 (54.4%) patients, with 102 (47.4%) patients having hs-cTn T ≥ 3× the upper limit of normal.

### 3.2 Angiographic indications and diagnoses

**Figure 1:**
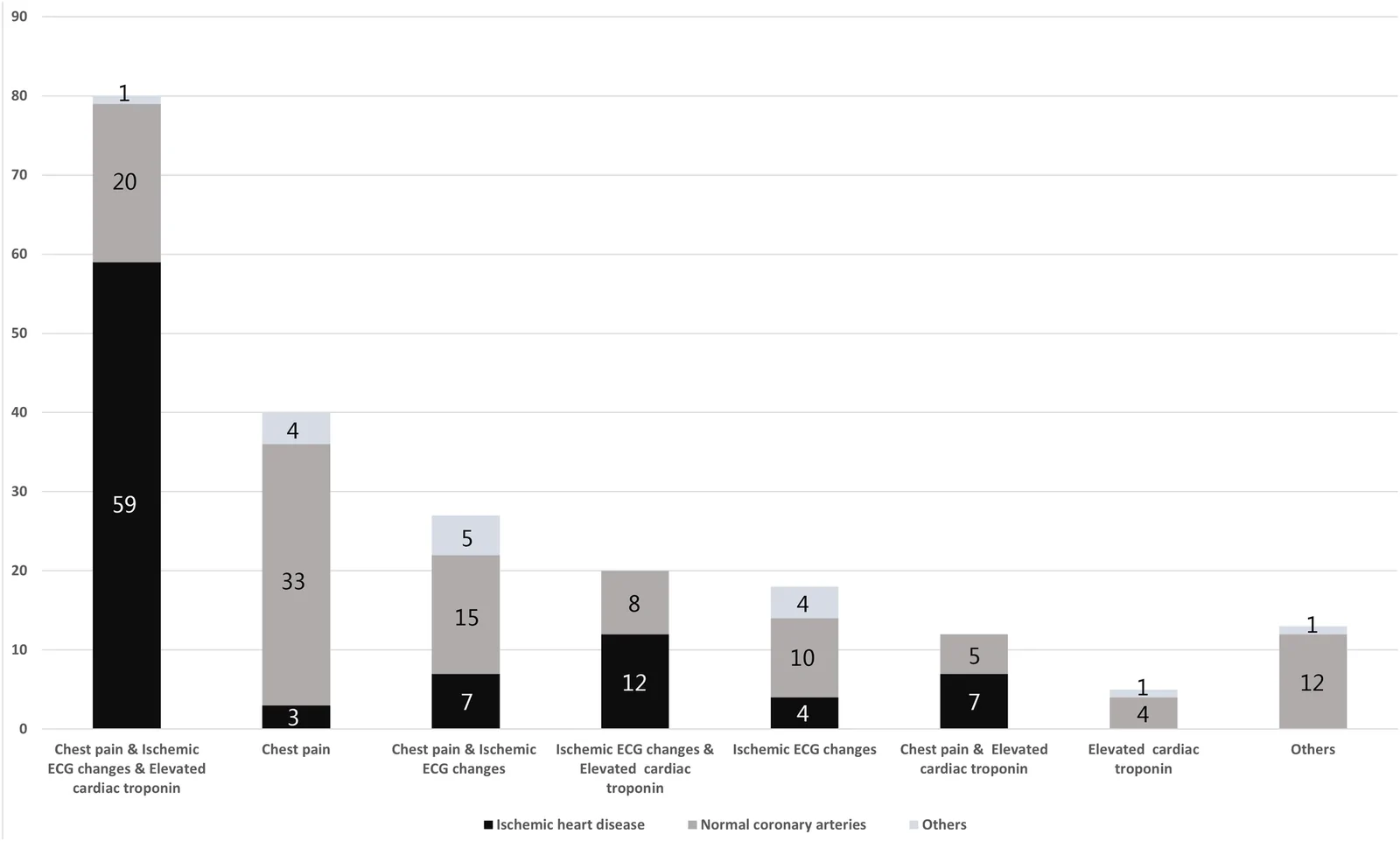
Angiographic indications and diagnoses.

The angiographic indications and final diagnoses are depicted in Figure 1. The majority of patients (94.0%) underwent CAG due to one or more of the following indications: chest pain, ischemic ECG changes, and/or elevated hs-cTn T levels. Notably, patients presenting with the combination of chest pain, ischemic ECG changes, and elevated hs-cTn T levels constituted the largest subgroup, making up 37.2% of the total patient population. Additional reasons for CAG included dyspnea and/or palpitations in 12 (5.6%) patients, as well as one case of suspected coronary artery fistula.

**Figure 2:**
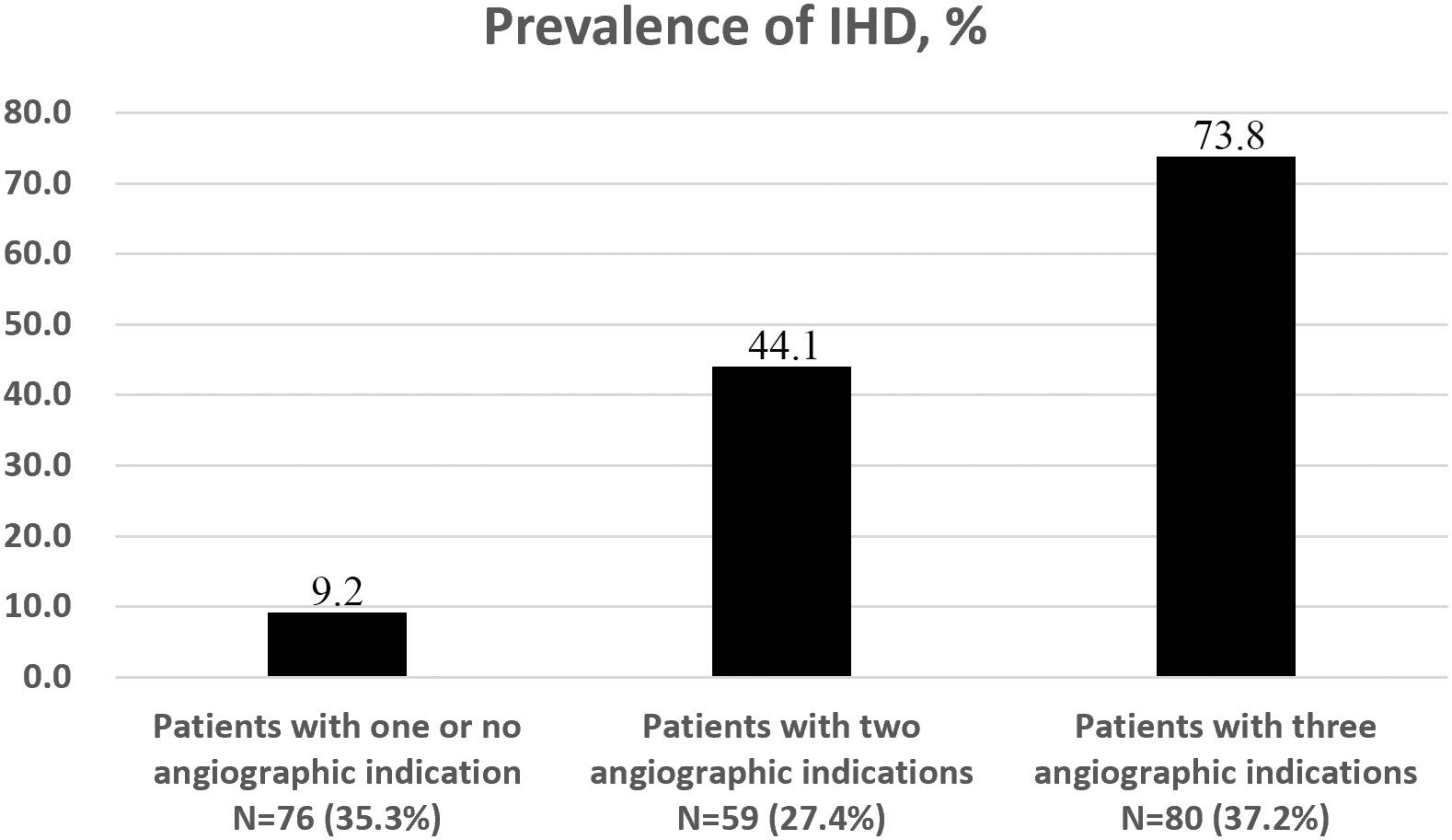
The prevalence of ischemic heart disease as determined by the number of angiographic indications.

In the cohort of 215 young patients undergoing CAG, 92 individuals (42.8%) were diagnosed with IHD, as detailed below. The prevalence of IHD was observed to be 9.2% (7 out of 76) in patients with one or no angiographic indications, 44.1% (26 out of 59) in those with two indications, and 73.8% (59 out of 80) in individuals with three indications (Figure 2). The distribution of IHD across these groups was statistically significant (*p* < 0.001). Subsequent pairwise comparisons among the groups (one or no indications vs. two indications, one or no indications vs. three indications, and two indications vs. three indications) demonstrated statistically significant differences, with all *p*-values being less than 0.001. Compared to patients with one or no angiographic indications, those with two indications exhibited significantly higher odds of IHD (OR, 7.8; 95% CI, 3.1–19.7), as did those with three indications (OR, 27.7; 95% CI, 11.0–69.7).

### 3.3 Coronary angiographic findings

The coronary angiographic findings for the 215 patients are presented in Table 2. Out of these patients, 107 (49.8%) had normal coronary arteries, while 92 (42.8%) exhibited various forms of ischemic heart disease, and 16 (7.4%) patients were diagnosed with mild coronary atherosclerosis and/or anomalies.

**Table 2:** Coronary angiographic findings.

Angiographic findings	Overall (*N* = 215)
Normal coronary arteries, *n* (%)	107 (49.8)
IHD, *n* (%)	92 (42.8)
Atherosclerotic IHD, *n* (%)	79 (36.7)
CAD	70 (32.6)
CAD & CAA/CAE	6 (2.8)
CAD & CAA/CAE &CAF	1 (0.5)
CAD & Severe coronary spasm	1 (0.5)
CAD & Severe myocardial bridging	1 (0.5)
Nonatherosclerotic IHD, *n* (%)	13 (6.0)
Severe myocardial bridging	3 (1.4)
Slow blood flow	3 (1.4)
Coronary thrombosis	2 (0.9)
Slow blood flow & CAF	1 (0.5)
Severe myocardial bridging & CAS	1 (0.5)
Severe coronary spasm	1 (0.5)
Severe coronary spasm & CAS	1 (0.5)
KD^a^	1 (0.5)
Others, n (%)	16 (7.4)
CAS	12 (5.6)
CAS & CAA/CAE	1 (0.5)
CAA/CAE	1 (0.5)
CAF	1 (0.5)
ALCAPA	1 (0.5)

IHD, ischemic heart disease; CAD, coronary artery disease; CAA, coronary artery aneurysm; CAE, coronary artery ectasia; CAF, coronary artery fistula; CAS, coronary atherosclerosis; KD, Kawasaki disease; ALCAPA, anomalous origin of the left coronary artery from the pulmonary artery.

a Causing bilateral coronary artery aneurysms and chronic total occlusion of the proximal left anterior descending coronary artery.

The final diagnoses for the 107 patients with normal coronary arteries are summarized in Table 3. In approximately one-fifth (23 out of 107) of these cases, the underlying cause of the symptoms remained largely unidentified despite undergoing a series of diagnostic evaluations. Among the 23 patients with unidentified etiologies, none exhibited elevated hs-cTn T levels. 16 patients presented with chest pain, 10 with palpitations, 9 with dyspnea and 8 with ischemic ECG changes. Echocardiographic assessments revealed mild cardiac chamber dilation, hypertrophy, and/or left ventricular systolic dyssynchrony in seven patients. Additionally, two patients had a history of ventricular septal defect closure. Notably, myocarditis (*n* = 21) and psychosomatic disease (generalized anxiety disorder=9, cardiac neurosis=3 and panic disorder=1) were the most common cardiac and noncardiac disorders, respectively. Other common noncardiac diagnoses were chest disease (pneumonia=1, pleurisy=1 and intramural hematoma of the aorta=1) and gastropathy (gastric ulcer=2 and erosive gastritis=1).

**Table 3:** Diagnoses of 107 patients with normal coronary arteries.

Diagnoses	Patients with normal coronary arteries (*N* = 107)
Unidentified causes, *n* (%)	23 (21.5)
Myocarditis, *n* (%)	21 (19.6)
Psychosomatic disease, *n* (%)	13 (12.1)
Cardiomyopathy, *n* (%)	10 (9.3)
Arrhythmia, *n* (%)	5 (4.7)
Congenital heart disease, *n* (%)	4 (3.7)
Chest disease, *n* (%)	3 (2.8)
Gastropathy, *n* (%)	3 (2.8)
Cardiac injury, *n* (%)	2 (1.9)
Valvular heart disease, *n* (%)	2 (1.9)
Others, *n* (%)	21 (19.6)

**Figure 3:**
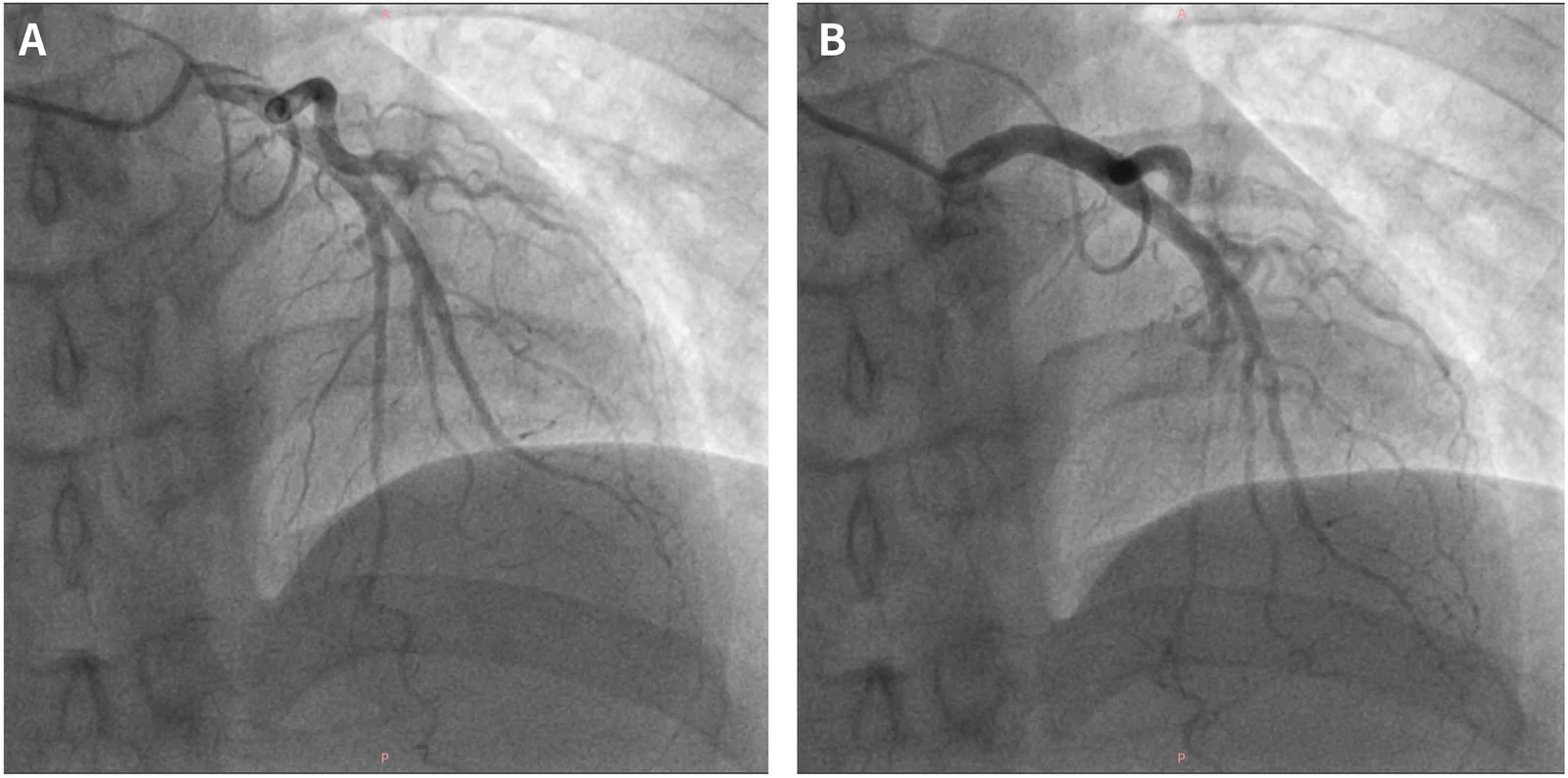
A 33-year-old male patient with severe myocardial bridging presenting with unstable angina pectoris. **(A)** Angiogram showing a normal left anterior descending coronary artery during diastole, but **(B)** severe stenosis of the middle left anterior descending coronary artery during systole.

**Figure 4:**
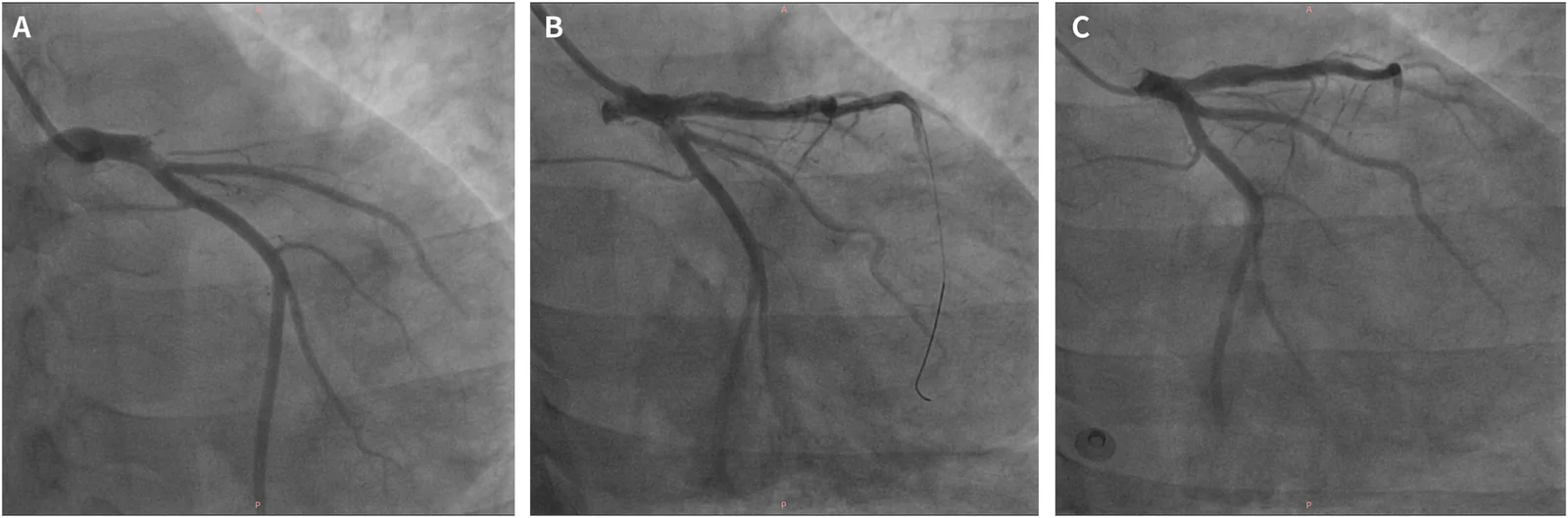
A 29-year-old male patient with hereditary thrombophilia exhibiting coronary thrombosis and acute ST-segment elevation myocardial infarction. **(A)** Initial angiogram revealing a large thrombus in the proximal left anterior descending coronary artery and left circumflex artery. **(B)** Angiogram following thrombus aspiration. **(C)** Angiogram 7 months later showing disappearance of coronary thrombus.

**Figure 5:**
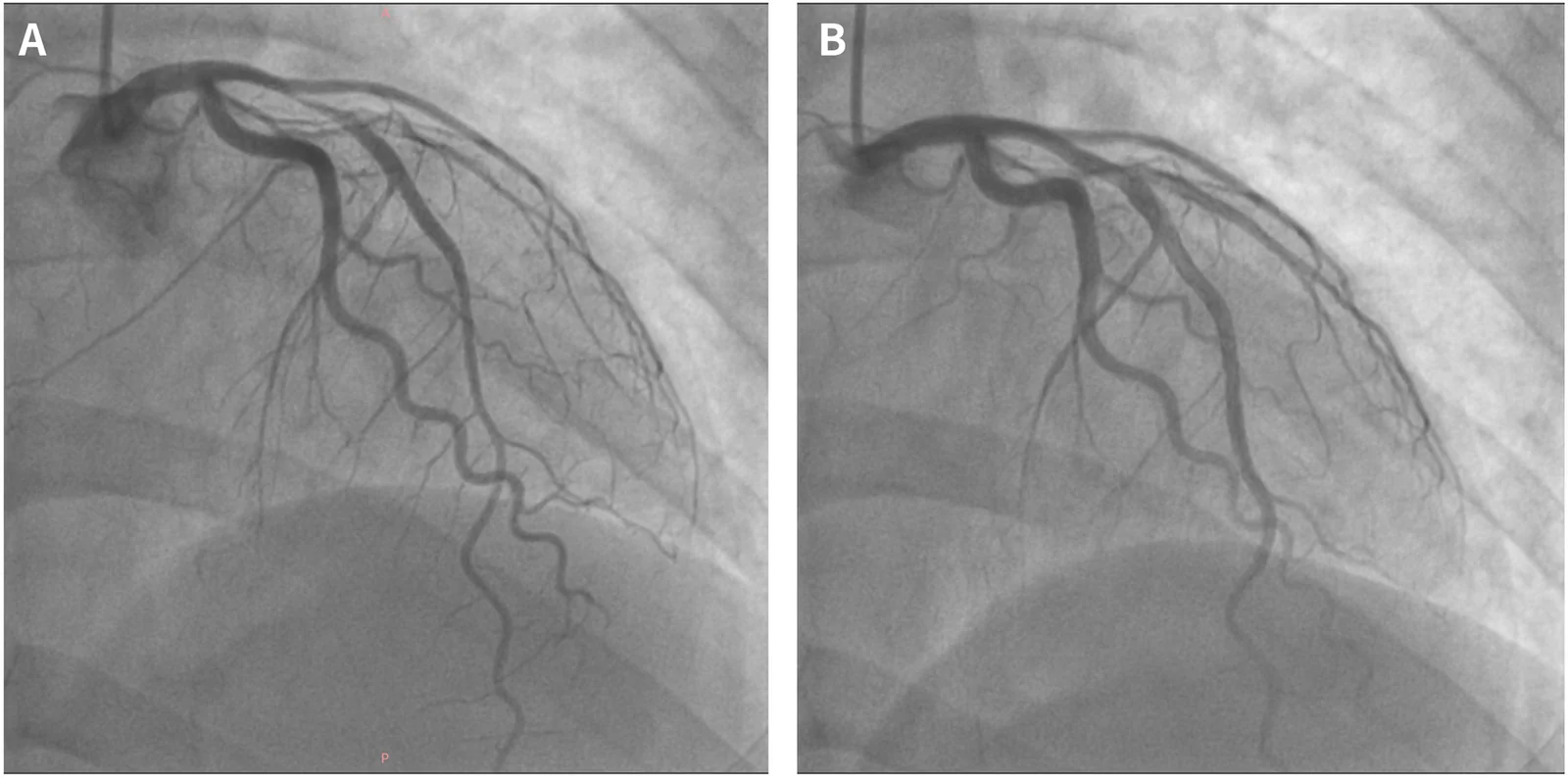
A 28-year-old male patient with severe coronary spasm manifesting as acute ST-segment elevation myocardial infarction. **(A)** Initial angiogram showing severe stenosis in the proximal left anterior descending coronary artery. **(B)** Angiogram following the intracoronary administration of nitroglycerin showing normalization of the affected left anterior descending coronary artery.

**Figure 6:**
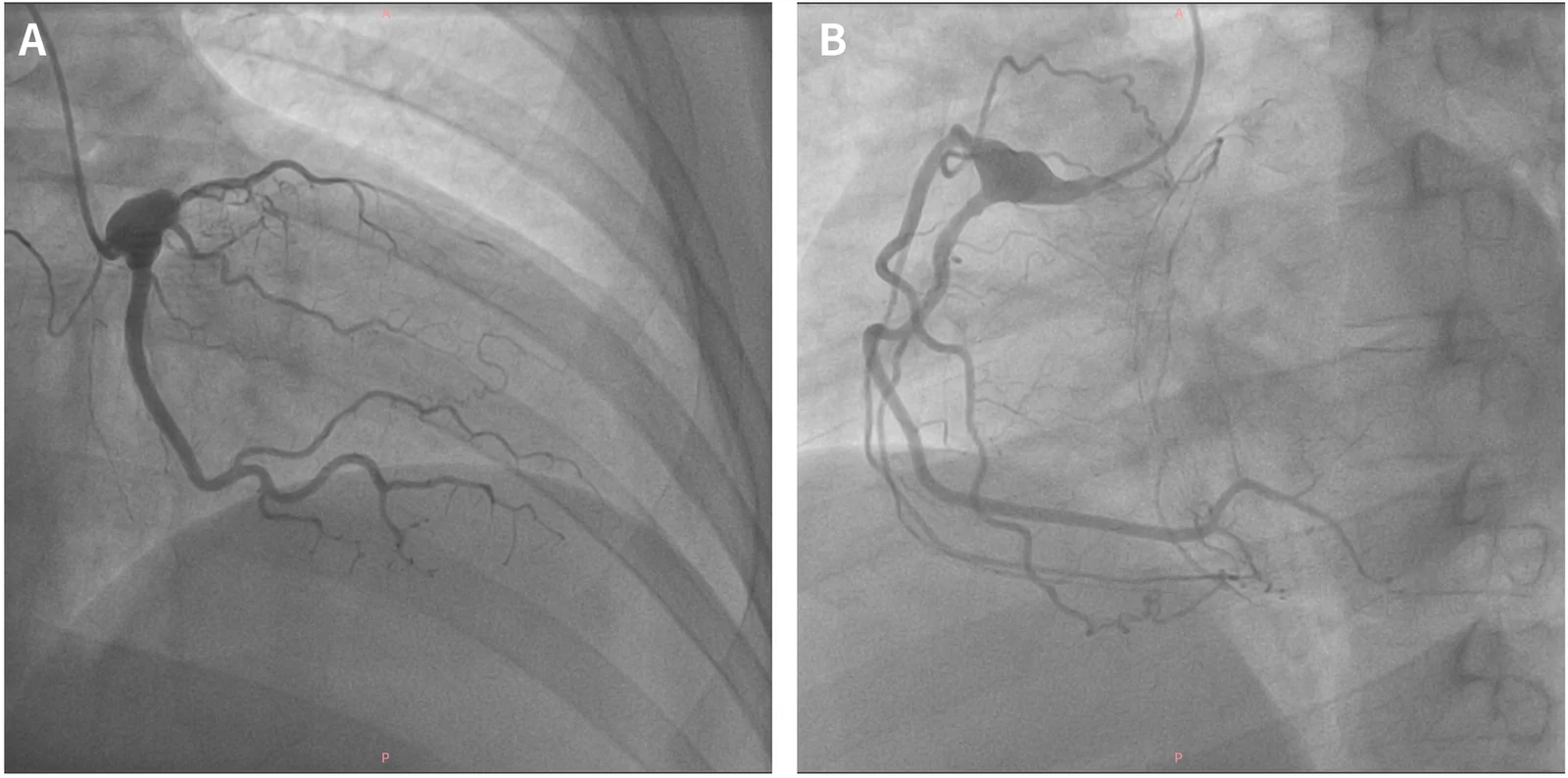
A 32-year-old male patient with kawasaki disease manifesting as bilateral coronary artery aneurysms **(A** and **B)** and chronic total occlusion of the proximal left anterior descending coronary artery **(A)**.

IHD was present in 92 (42.8%) patients and encompassed a spectrum of atherosclerotic and nonatherosclerotic conditions. While atherosclerosis was identified as the predominant cause of IHD, nonatherosclerotic factors such as myocardial bridging, slow coronary blood flow, coronary thrombosis, coronary spasm, and Kawasaki disease also played significant roles (Figure 3–6). Additionally, 72 (33.5%) patients presented with myocardial infarction with obstructive coronary artery disease [63 STEMI, 9 non-ST segment elevation myocardial infarction (NSTEMI)] and 5 (2.3%) patients manifested as myocardial infarction with non-obstructive coronary arteries (MINOCA: 2 STEMI, 3 NSTEMI). Moreover, 37/77 (48.1%) patients with AMI had coronary multivessel disorders.

In this study, 10/215 (4.7%) patients had coronary artery aneurysms/ectasias (CAAs/CAEs). They had 1-5 traditional ASCVD risk factors, 7/10 (70.0%) patients had significant CAD, and 6/10 (60.0%) patients were diagnosed with coronary multivessel disease. Based on their illness history, angiographic features, chest computed tomography and echocardiographic findings, 8/10 (80.0%) patients' CAAs/CAEs were probably caused by atherosclerosis, while the other 2/10 (20.0%) patients' CAAs/CAEs probably resulted from Kawasaki disease and congenital anomaly, respectively.

Additionally, among the 4 (1.9%) patients with coronary slow flow, all patients manifested as chest pain, 3 patients had elevated hs-cTn T levels, and 2 patients presented with ischemic ECG changes. Furthermore, the 3 patients with severe coronary spasm exhibited chest pain or dyspnea, elevated ST segment and elevated hs-cTn T levels, which led to the diagnosis of STEMI. Lastly, isolated coronary atherosclerosis was not uncommon and affected 12 (5.6%) patients.

### 3.4 Comparison of patient characteristics between patients with and without ischemic heart disease

In comparison to patients without IHD, those with IHD were more likely to present with chest pain (82.6% versus 67.5%), a history of diabetes mellitus (16.3% versus 2.4%), ischemic ECG changes (89.1% versus 51.2%), a thicker interventricular septum [10 (10-11) mm versus 10 (9-11) mm], and higher median peak hs-cTn T levels (1.253 pg/mL versus 0.007 pg/mL) (Table 4). Conversely, patients with IHD were less likely to have arrhythmia (4.3% versus 16.3%), and exhibited lower median peak NT-pro BNP levels (100 pg/mL versus 153 pg/mL).

**Table 4:** Comparison of patient characteristics between very young patients with and without ischemic heart disease.

Characteristics	Patients with IHD (*N* = 92)	Patients without IHD (*N* = 123)	*P*-value
Age, y	32 (30–34)	32 (28–34)	0.73
Male, *n* (%)	77 (83.7)	104 (84.6)	0.87
BMI, Kg/m^2^	26.2 (23.0–29.1)	24.7 (21.7–27.3)	0.14
Smoking, pack.year	0 (0–10)	2 (0–10)	0.32
Clinical manifestations, *n* (%)			
Chest pain	76 (82.6)	83 (67.5)	0.01
Dyspnea	50 (54.3)	66 (53.7)	0.92
Palpitation	26 (28.3)	42 (34.1)	0.36
Comorbidity, *n* (%)			
Hypertension	24 (26.1)	26 (21.1)	0.40
Diabetes mellitus	15 (16.3)	3 (2.4)	＜0.01
Valvular heart disease	3 (3.3)	4 (3.3)	1.00
Prior coronary artery disease	7 (7.6)	0 (0)	0.002
Electrocardiographic findings, *n* (%)			
Ischemic ECG changes	82 (89.1)	63 (51.2)	＜0.01
ST-segment elevation	63 (68.5)	26 (21.1)	＜0.01
T-wave inversion	32 (34.8)	36 (29.3)	0.39
Pathological Q waves	43 (46.7)	9 (7.3)	＜0.01
ST-segment depression	21 (22.8)	14 (11.4)	0.03
Arrhythmia	4 (4.3)	20 (16.3)	0.01
Echocardiographic findings			
LA dimension, mm	32 (30–34)	31 (28–37)	0.27
LV dimension, mm	48 (45–51)	47 (44–52)	0.66
RA dimension, mm	42 (39–45)	42 (40–46)	0.50
RV dimension, mm	20 (19–22)	20 (19–23)	0.35
IVS thickness, mm	10 (10–11)	10 (9–11)	0.03
LVPW thickness, mm	10 (9–10)	9 (9–10)	0.08
LVEF, %	65 (59–67)	63 (57–66)	0.06
Laboratory tests			
Hemoglobin, g/L	152 (143–163)	150 (138–160)	0.28
LDL-C, mmol/L	2.9 (2.3–3.6)	2.8 (2.1–3.4)	0.27
Serum creatinine, mmol/L	71 (63–83)	69 (59–79)	0.16
Serum uric acid, mmol/L	420 ± 119	408 ± 136	0.51
Peak NT-pro BNP, pg/mL	100 (36–270)	153 (100–632)	0.002
Elevated hs-cTn T, *n* (%)	78 (84.8)	39 (31.7)	＜0.01
Peak hs-cTn T, pg/mL	1.253 (0.057–4.260)	0.007 (0.005–0.072)	＜0.01

IHD, ischemic heart disease; BMI, body mass index; ECG, electrocardiogram; LA, left atrium; LV, left ventricle; RA, right atrium; RV, right ventricle; IVS, interventricular septum; LVPW, left ventricular posterior wall; LVEF, left ventricular ejection fraction; LDL-C, low-density lipoprotein cholesterol; NT-pro BNP, N-terminal pro-B-type natriuretic peptide; hs-cTn T, high-sensitivity cardiac troponin T.

### 3.5 Predictors of ischemic heart disease

In the univariate logistic regression analysis for IHD, the following variables were identified as significant determinants: chest pain, diabetes mellitus, ischemic ECG changes, arrhythmia, interventricular septum thickness, left ventricular ejection fraction, the logarithm of peak NT-pro BNP, and elevated hs-cTn T levels (Table 5). Subsequent multivariate logistic regression analysis revealed that diabetes mellitus (OR, 14.71; 95% CI, 1.59-135.76; *p* = 0.018), ischemic ECG changes (OR, 3.11; 95% CI, 1.09-8.91; *p* = 0.034), interventricular septum thickness (OR, 1.46; 95% CI, 1.08-1.98; *p* = 0.015), and elevated hs-cTn T levels (OR, 8.26; 95% CI, 3.17-21.51; *p* < 0.001) were independent predictors of IHD.

**Table 5:** Predictors of ischemic heart disease in univariable and multivariable logistic regression analysis.

Characteristics	Univariate analysis		Multivariate analysis	
	OR (95% CI)	*p*	OR (95% CI)	*P*
Age, y	1.05 (0.99–1.13)	0.13		
Male	0.94 (0.45–1.96)	0.87		
BMI, Kg/m^2^	1.04 (0.97–1.12)	0.30		
Smoking, pack.year	0.98 (0.95–1.02)	0.39		
Chest pain	2.29 (1.19–4.42)	0.014	1.91 (0.69–5.31)	0.21
Dyspnea	1.03 (0.60–1.77)	0.92		
Palpitation	0.76 (0.42–1.37)	0.36		
Hypertension	1.32 (0.70–2.49)	0.40		
Diabetes mellitus	7.79 (2.18–27.81)	0.002	14.71 (1.59–135.76)	0.018
Ischemic ECG changes	7.81 (3.71–16.46)	＜0.001	3.11 (1.09–8.91)	0.034
Arrhythmia	0.23 (0.08–0.71)	0.01	0.14 (0.01–1.39)	0.09
LA dimension, mm	1.01 (0.95–1.07)	0.82		
LV dimension, mm	0.99 (0.95–1.03)	0.65		
RA dimension, mm	0.98 (0.93–1.03)	0.42		
RV dimension, mm	0.93 (0.81–1.06)	0.27		
IVS thickness, mm	1.25 (1.04–1.51)	0.018	1.46 (1.08–1.98)	0.015
LVPW thickness, mm	1.19 (0.93–1.54)	0.17		
LVEF, %	1.04 (1.00–1.08)	0.043	1.03 (0.96–1.09)	0.44
LDL-C, mmol/L	1.07 (0.89–1.29)	0.47		
Serum creatinine, mmol/L	1.00 (1.00–1.00)	0.66		
Serum uric acid, mmol/L	1.00 (1.00–1.00)	0.51		
lg peak NT-pro BNP	0.57 (0.37–0.88)	0.012	0.72 (0.37–1.42)	0.350
Elevated hs-cTn T	12.00 (6.06–23.78)	＜0.001	8.26 (3.17–21.51)	＜0.001

BMI, body mass index; ECG, electrocardiogram; LA, left atrium; LV, left ventricle; RA, Right atrium; RV, right ventricle; IVS, interventricular septum; LVPW, left ventricular posterior wall; LVEF, left ventricular ejection fraction; LDL-C, low-density lipoprotein cholesterol; NT-pro BNP, N-terminal pro-B-type natriuretic peptide; hs-cTn T, high-sensitivity cardiac troponin T.

**Figure 7:**
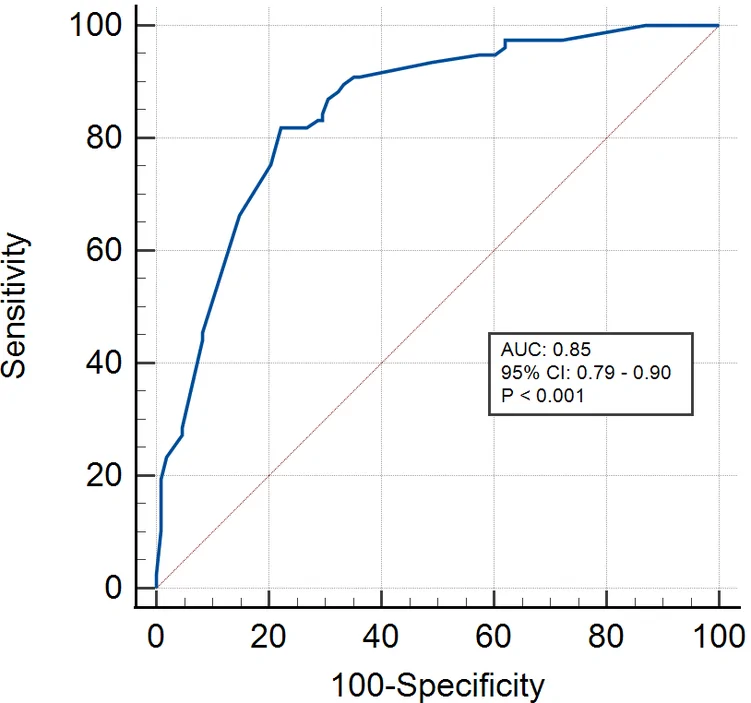
Receiver operating characteristic curves to distinguish patients with and without ischemic heart disease.

To assess the predictive performance of the multivariable model, ROC curve analysis was performed using the predicted probabilities from the combined logistic regression model. The combined model showed a robust discriminative ability for predicting IHD, with an AUC of 0.85 (95% CI, 0.79-0.90; *p* < 0.001) (Figure 7).

## 4 Discussion

This study presents several important findings. Firstly, within the highly selected cohort of 215 young patients, traditional ASCVD risk factors were very common, with notably high prevalence rates of IHD and mild coronary atherosclerosis. Secondly, the majority of patients underwent CAG due to chest pain, ischemic ECG changes, and/or elevated hs-cTn T levels. The presence of two or more of these indications was associated with an increased risk of IHD. Thirdly, diabetes mellitus, ischemic ECG changes, interventricular septum thickness, and elevated hs-cTn T levels were identified as independent predictors of IHD. The combination of these factors demonstrated a favorable diagnostic value for IHD. Fourthly, the majority of IHD cases were observed in male patients, primarily attributed to coronary atherosclerosis, and typically manifested as STEMI and single-vessel disease. Lastly, nonatherosclerotic causes of IHD, including coronary slow flow, severe myocardial bridging, severe coronary spasm, hereditary thrombophilia, and Kawasaki disease, were not uncommon.

ASCVD is one of the leading causes of death worldwide (1). Although ASCVD is more prevalent among older populations, recent decades have witnessed a troubling rise in premature AMI (2). Traditional risk factors for ASCVD—such as hypertension, diabetes mellitus, dyslipidemia, obesity, smoking, and sedentary behavior—are increasingly affecting young adults and even children (2–4). Additionally, emerging lifestyle factors, including excessive stress, sleep disorders, prolonged television viewing, and poor dietary quality, have been identified as new risk factors (5–8). Consequently, the onset of ischemic heart disease is occurring at increasingly younger ages. Consistent with previous research (16, 17), our study revealed that the majority of patients (78.1%) presented with at least one traditional ASCVD risk factor. Among these, obesity/overweight, smoking, dyslipidemia, hypertension, and diabetes mellitus are probably associated with unhealthy lifestyles and are potentially modifiable. Notably, emerging studies have highlighted the significant role of uric acid in processes such as oxidative stress, inflammation, and the proliferation of vascular smooth muscle cells, all of which are integral to the pathogenesis of atherosclerosis (18). In our cohort, hyperuricemia was present in 92 (42.8%) patients, making it one of the most prevalent risk factors identified. The association between ASCVD risk factors and the development of coronary atherosclerosis, as well as subsequent CAD, has been extensively documented (19–22). The substantial burden of ASCVD risk factors, alongside the high prevalence of mild coronary atherosclerosis (7.0%) and CAD (36.7%) observed in our study, underscore the critical need for lifestyle modifications and effective risk factor management.

Very young patients with CAD are distinct and uncommon demographic, comprising approximately 6% to 6.5% of those with premature CAD (17, 23). The consequences of missed or misdiagnosed premature AMI can be significant both clinically and socially. According to Victor's study, CAD is the leading cause of sudden cardiac arrest in adults under 40 years old (24). However, the rates of immediate coronary angiography are low, resulting in missed opportunities for timely intervention (24). In our cohort of 215 patients, the majority underwent coronary angiography due to one or more of the following indications: chest pain, ischemic ECG changes, and/or elevated hs-cTn T levels. Notably, patients presenting with all three angiographic indications constituted over one-third of the cohort, with 73.8% ultimately diagnosed with IHD, most commonly manifesting as STEMI and requiring emergency percutaneous coronary intervention. Conversely, patients presenting with one or none of the angiographic indications were more frequently diagnosed with conditions unrelated to IHD. Therefore, the presence of two or three of the aforementioned angiographic indications should elevate the clinical suspicion of IHD.

It is important to recognize that conditions such as myocarditis, cardiomyopathy, psychosomatic disorders, gastropathy, and various other noncardiac illnesses can closely mimic IHD, thereby presenting substantial diagnostic challenges and complicating treatment strategies (14, 25). In this setting, the integration of comprehensive history-taking, thorough physical examination, intravascular imaging, cardiac magnetic resonance imaging, endomyocardial biopsy, and collaboration among multidisciplinary teams is essential for achieving accurate diagnosis and effective management (14, 26). Nonetheless, the potential for missed, incorrect, or delayed diagnoses of premature IHD persists. Our study identified diabetes mellitus, ischemic ECG changes, interventricular septum thickness, and elevated hs-cTn T levels as independent predictors of IHD. The combined multivariable model demonstrated a robust discriminative performance (AUC = 0.85; 95% CI = 0.79-0.90; *p* < 0.001). Consequently, in cases of suspected IHD, these variables may enhance diagnostic accuracy and facilitate differential diagnosis.

As expected, the majority of IHD patients in our study were male, with the condition predominantly arising from coronary atherosclerosis, typically manifesting as obstructive STEMI and single-vessel disease—findings that align with prior research (12, 16, 27). Nevertheless, nonatherosclerotic coronary artery disorders, including severe myocardial bridging, slow coronary blood flow, severe coronary spasm, coronary thrombosis, and Kawasaki disease, were also observed, accounting for 14.1% of IHD cases. Additionally, we identified rare congenital coronary anomalies, such as coronary artery fistula and the anomalous origin of the left coronary artery from the pulmonary artery.

Consistent with previous studies, the prevalence of MINOCA among our AMI patients was 6.5% (28). Typically, MINOCA is more prevalent in females, with coronary atherosclerosis being identified as the primary cause (29). Interestingly, all five MINOCA patients in our study were male and active smokers; two cases were attributed to severe coronary spasm leading to STEMI (Figure 5), while the remaining three were caused by coronary slow flow, resulting in NSTEMI. This discrepancy may be attributed to variation in study population and design. Our cohort was relatively small and predominantly comprised younger males, which introduced a significant selection bias. Furthermore, the absence of routine intravascular imaging meant that many coronary plaques were not thoroughly evaluated. Notably, patients with coronary slow flow frequently experience chest pain and often require hospital readmission. Furthermore, they are at an increased risk for complications such as myocardial infarction, heart failure, ventricular arrhythmias, and sudden cardiac death (15). A recent cohort study with a seven-year follow-up period demonstrated that individuals with coronary slow flow exhibit a higher incidence of major adverse cardiovascular events and recurrent hospitalizations (30). Additionally, the presence of coronary slow flow is associated with an increased risk of adverse events and serves as an independent predictor of clinical outcomes in patients with MINOCA, as evidenced by an adjusted hazard ratio of 2.76 (95% CI, 1.34–5.67; *P* = 0.006) (31). Collectively, these findings suggest that coronary slow flow is not a benign condition, underscoring the necessity for close follow-up to enhance overall survival.

Thrombophilia plays a significant role in the pathogenesis of acute myocardial infarction, especially among young patients with fewer cardiovascular risk factors and less atherosclerotic burden (32). In a retrospective study involving 46 consecutive young patients with STEMI aged ≤ 35 years, overt hypercoagulable state was detected serologically (antiphospholipid antibodies syndrome) or clinically (left ventricular or coronary thrombosis without an atherosclerotic plaque) in 2 (4.3%) and 6 (13.0%) patients, respectively (23). Interestingly, through the application of whole exome gene sequencing, we identified 2 (0.9%) patients with hereditary thrombophilia who presented with STEMI and coronary thrombosis (Figure 4). As routine screening for thrombophilia was not conducted, the actual prevalence of this condition among our patient cohort may be underestimated. Therefore, it is essential to screen for thrombophilia in very young STEMI patients with fewer ASCVD risk factors and less atherosclerotic burden.

In our study, the prevalence of CAAs/CAEs was 4.7% with coronary atherosclerosis identified as the primary cause, which was consistent with previous report (33). However, the estimated prevalence of Kawasaki disease-related CAAs/CAEs in young adults with acute coronary syndrome (ACS) is 5%-7.4% (34, 35), which is significantly higher than what we reported. In the current study, we identified one case of asymptomatic CAAs associated with Kawasaki disease (Figure 6). This case was characterized by the presence of pathological Q waves and persistent ST-segment elevation in leads V1–V5, left ventricular dilation and dysfunction, apical aneurysm, bilateral calcified CAAs, and chronic total occlusion of the proximal left anterior descending coronary artery (36). The observed discrepancy may be attributed to differences in the study populations, as Shyu's study included older participants with ACS and elevated low-density lipoprotein levels (34). CAAs/CAEs can result from both coronary atherosclerosis and Kawasaki disease. The former typically affects older individuals and is often associated with atherosclerotic plaques in multiple vessels (37). In contrast, CAAs/CAEs resulting from Kawasaki disease generally involve the proximal segments of the coronary arteries and has fewer ASCVD risk factors (37). Consequently, Kawasaki disease should be considered as a potential, albeit uncommon, cause of IHD and CAAs/CAEs, particularly in younger patients presenting with multiple, proximal, and calcified CAAs/CAEs.

Lastly, a substantial proportion of patients with normal coronary arteries exhibit unidentified etiologies despite undergoing a series of diagnostic evaluations. The high prevalence of chest pain, ischemic ECG changes, and abnormal echocardiographic findings may suggest the presence of atypical forms of IHD, such as coronary spasm and coronary microvascular dysfunction. In such cases, provocative testing with intracoronary acetylcholine and/or invasive physiological assessments during CAG should be considered (38). Additionally, some patients with psychosomatic disorders, such as generalized anxiety disorder and cardiac neurosis, may present with cardiac symptoms and a strong propensity for seeking medical care, despite the absence of underlying cardiac pathology. Therefore, increased attention should be directed towards these under-researched disorders, and psychiatric consultation should be sought when necessary, particularly in younger patients.

## 5 Limitations

This study has several limitations. Firstly, it is a single-center, retrospective investigation, making selection bias unavoidable. Secondly, due to the severity of many patients' conditions, advanced imaging techniques such as intravascular imaging or cardiac magnetic resonance were not routinely utilized. Thirdly, we did not conduct provocative testing with intracoronary acetylcholine or ergonovine, potentially overlooking cases of coronary spasm. Lastly, the absence of routine thrombophilia screening among our patients may lead to an underestimation of its prevalence.

## 6 Conclusions

The present study demonstrated a significant prevalence of modifiable atherosclerotic risk factors among very young patients undergoing CAG. Although atherosclerosis was identified as the primary cause of IHD, nonatherosclerotic etiologies also contributed substantially. The presence of two or more angiographic indications—namely, chest pain, ischemic ECG changes, and/or elevated hs-cTn T levels—was associated with an increased risk of IHD. Furthermore, diabetes mellitus, ischemic ECG changes, interventricular septum thickness, and elevated hs-cTn T levels were independent predictors of premature IHD.

## Data Availability

The original contributions presented in the study are included in the article/Supplementary Material, further inquiries can be directed to the corresponding authors.
